# EMPOWERING COLLABORATION: ENHANCING GERIATRIC CARE WITH AGE-FRIENDLY CNA TRAINING BY LTC OMBUDSMEN

**DOI:** 10.1093/geroni/igae098.4289

**Published:** 2024-12-31

**Authors:** Theresa Sivers-Teixeira, Erin Thayer, E-Shien Chang, Stella Chen, Debra Cherry, Rachel Tate, Bonnie Olsen

**Affiliations:** Keck School of Medicine of University of Southern California, Alhambra, California, United States; Keck School of Medicine of USC, University of Southern California, Department of Family Medicine, Keck School of Medicine of USC, University of Southern California, California, United States; Weill Cornell Medical College, New York, New York, United States; Keck School of Medicine, Los Angeles, California, United States; Alzheimer’s Los Angeles, Los Angeles, CA 90010, California, United States; Wise and Healthy Aging, Santa Monica, California, United States; University of Southern California, Los Angeles, California, United States

## Abstract

Long-Term Care (LTC) Certified Nursing Assistants (CNAs) who provide direct care to residents often face significant challenges in supporting daily activities and the well-being of residents with functional decline. Frequent difficult behaviors can lead to negative experiences, reducing residents’ quality of life and diminishing CNAs’ job satisfaction. LTC Ombudsmen (LTCO) assist residents with issues related to day-to-day care, health, safety, personal preferences, rights violations, poor quality of care, and elder abuse. The USC Geriatric Workforce Enhancement Program (GWEP) developed an innovative program to train LTCO to deliver the Age-Friendly (AF) Nursing Home curriculum to improve CNAs’ geriatric competencies. A four-lesson curriculum, developed collaboratively by the Los Angeles LTCO Program administered by WISE & Healthy Aging, Alzheimer’s Los Angeles, Dr. Shien Chang, and the USC GWEP team, addresses: 1) AF principles of nursing home care, 2) evidence-based strategies for managing behavior issues related to dementia, and 3) strategies for increasing culturally appropriate communication with patients and caregivers. Using a train-the-trainer model, 5 LTCOmbudsmen were trained and delivered the curriculum to 133 CNAs in eight local nursing homes. A pre-post training survey administered to CNA participants showed a statistically significant increase in confidence in applying the training topics. Focus groups with LTCO trainers revealed a positive bidirectional impact on ombudsmen and CNAs. This training model introduced AF care strategies and strengthened the shared purpose between LTCO and CNAs. The study provides a foundation for ongoing collaboration and dissemination of this scalable and sustainable model to improve care for older adult LTC residents.

